# Assessing the Impact of a Risk-Based Intervention on Piped Water Quality in Rural Communities: The Case of Mid-Western Nepal

**DOI:** 10.3390/ijerph15081616

**Published:** 2018-07-31

**Authors:** Dorian Tosi Robinson, Ariane Schertenleib, Bal Mukunda Kunwar, Rubika Shrestha, Madan Bhatta, Sara J. Marks

**Affiliations:** 1 Eawag, Swiss Federal Institute of Aquatic Science and Technology, Überlandstrasse 133, 8600 Dübendorf, Switzerland; dorian.tosirobinson@gmail.com (D.T.R.); ariane.schertenleib@eawag.ch (A.S.); 2 Helvetas Swiss Intercooperation Nepal, Jhamshikhel Dhobi Ghat, Lalitpur, GPO Box 688 Kathmandu, Nepal; Bal.Kunwar@helvetas.org (B.M.K.); Rubika.Shrestha@helvetas.org (R.S.); Madan.Bhatta@helvetas.org (M.B.)

**Keywords:** *E. coli*, monitoring, drinking water, water safety plan, sanitary inspection, gravity-fed piped water scheme, risk management

## Abstract

Ensuring universal access to safe drinking water is a global challenge, especially in rural areas. This research aimed to assess the effectiveness of a risk-based strategy to improve drinking water safety for five gravity-fed piped schemes in rural communities of the Mid-Western Region of Nepal. The strategy was based on establishing community-led monitoring of the microbial water quality and the sanitary status of the schemes. The interventions examined included field-robust laboratories, centralized data management, targeted infrastructure improvements, household hygiene and filter promotion, and community training. The results indicate a statistically significant improvement in the microbial water quality eight months after intervention implementation, with the share of taps and household stored water containers meeting the international guidelines increasing from 7% to 50% and from 17% to 53%, respectively. At the study endline, all taps had a concentration of <10 CFU *Escherichia coli*/100 mL. These water quality improvements were driven by scheme-level chlorination, improved hygiene behavior, and the universal uptake of household water treatment. Sanitary inspection tools did not predict microbial water quality and, alone, are not sufficient for decision making. Implementation of this risk-based water safety strategy in remote rural communities can support efforts towards achieving universal water safety.

## 1. Introduction

In recent years, water sector professionals have made considerable progress improving access to drinking water worldwide. The Millennium Development Goal (MDG) for drinking water was met in 2015, with 2.6 billion people gaining access to an improved drinking water source since 1990 [1]. However, the additional sanitary protection offered by an improved drinking water source does not ensure that the water is safe to drink, because it is not guaranteed to be free from fecal contamination [2,3]. Half a million people worldwide died in 2012 due to consumption of unsafe water [4]. The MDGs thus underscored an urgent need to prioritize interventions designed to limit the hazards to human health by meeting the international guidelines for drinking water safety [5].

To address this issue, the water sector adopted Sustainable Development Goal (SDG) 6, which now includes measures of availability, accessibility, and quality as core standards in its definition of safely managed drinking water [6]. With these considerations, over a quarter of the global population currently lacks access to safely managed drinking water [7]. Water sector practitioners, therefore, face the challenging objective to deliver “universal and equitable access to safe and affordable drinking water for all by 2030” (SDG 6.1).

In Nepal, only a quarter of the rural population was estimated to have access to safely managed drinking water in 2015 [7], with access rates being lowest in the most remote areas where treatment is virtually non-existent and microbial contamination of water supplies is well documented. For example, Shrestha et al. (2017) reported inadequate water, sanitation, and hygiene (WASH) conditions in rural Nepal [8]. In the hilly areas of Mid-Western Nepal, a previous study reported a high health risk associated with the consumption of water from public taps, with 69% of samples collected testing positive for *Escherichia coli (E. coli)*. One in ten samples contained more than 100 colony forming units (CFU) of *E. coli*/100 mL [9], considered at very high risk per World Health Organization (WHO) classifications [5]. Another study in this region reported high daily variability and peak concentrations of fecal contamination [10].

These studies indicate a need for a comprehensive risk management strategy in place of end-of-pipe testing. Shrestha et al. (2017) recommended regular monitoring of water quality to generate missing information regarding seasonal variations [8]. The authors additionally suggested several mitigation actions, such as source protection, regular inspections, and targeted upgrades, with an emphasis on community engagement and water treatment measures. Such activities align with the World Health Organization (WHO)’s Water Safety Plan (WSP) approach that has been (and continues to be) widely promoted for improving drinking water safety from the source to the consumer. This approach is based on the identification of hazards and the mitigation of risks to achieve multibarrier protections for public health safety [5]. WSPs can be adapted to the needs of any drinking water project, including small communities’ water supplies [11,12]. One tool used in small community WSPs is the sanitary inspection form to systematically assess vulnerabilities throughout the water scheme. These assessment forms proactively identify hazards at critical locations, thereby informing the management team regarding the potential sources of contamination to the water system and the mitigation efforts required.

While the WSP approach supports operational management processes for drinking water supplies, String and Lantagne point to a need for “evidence-based, documented impacts to both water supply and health after WSP implementation” [13]. Evidence regarding the implementation and impacts of WSPs on water quality is especially lacking in remote rural settings, where monitoring activities are hindered by low access to laboratory resources and technical expertise. Additionally, the suitability of sanitary inspection tools for assessing water safety is questioned, with previous studies showing contradictory conclusions regarding the predictability of fecal pollution levels based on sanitary risk scores alone [14,15,16]. It is therefore argued that effective risk management for water supplies should combine sanitary protection indicators with regular water quality testing [3,16]. In addition, the WHO has developed a revised set of forms that better suit the reality of small water supplies in rural contexts [17].

The objective of this research was to describe and evaluate a risk-based water safety strategy within five rural communities served by gravity-fed piped water supply schemes in the Dullu municipality in Mid-Western Nepal. Using a controlled before-and-after study design, we assessed the impact of a suite of interventions on the microbial water quality at different points throughout the system over an eight-month period. The interventions included the reinforcement of a pre-existing household water treatment and safe storage (HWTS) promotion campaign and targeted infrastructural and management improvements to the water schemes. Regular water quality monitoring was established using two solar-powered field laboratories equipped for microbial testing, and adapted sanitary inspections tools were used to systematically assess risks to the water systems. Intensive community participation and training were core features throughout the project’s implementation.

In addition to the main objective of evaluating intervention impacts on the microbial water quality, other research questions of interest were as follows: (1) How did community members engage with the risk management process for their water system? (2) To what extent were the water safety interventions taken up by the communities by the study endline? (3) Did sanitary inspection scores align with water quality testing results? The project was implemented by Helvetas Swiss Intercooperation Nepal’s (hereafter referred to as Helvetas-Nepal) Integrated Water Resources Management (IWRM) program, in collaboration the Swiss Federal Institute of Aquatic Science and Technology (hereafter referred to as Eawag) and REACH: Improving Water Security for the Poor (a program led by Oxford University and funded by the United Kingdom (UK) Government). The study commenced with baseline data collection from 120 households across five intervention communities and three control communities. To assess outcomes, an endline assessment was performed eight months after the baseline to capture changes in the microbial water quality and in households’ perceptions and behavior regarding their drinking water.

The water safety strategy showed promising results towards achieving SDG 6.1 in rural communities dependent on gravity-fed piped schemes. Within intervention communities, we observed water quality improvements at taps and within households, improved hygiene behavior, and increased community capacity to proactively identify and mitigate the risks identified through regular monitoring. However, the microbial water quality did not meet the international guidelines by the study’s endline for 100% of the water points assessed, indicating that further efforts are needed to ensure universal access to safe drinking water in this setting. This study also revealed the limitations of sanitary inspection scores and concluded that such tools should be combined with regular water quality testing for a complete risk management approach.

## 2. Materials and Methods

### 2.1. Study Site

Nepal is a landlocked country in Southern Asia that is situated in the Himalayas and shares borders with India and China. Three main regions compose the country’s landscape and climate: a flat tropical area called the Terai, an intermediate hilly region, and the Himalayan mountains [18]. In 2017, the population was estimated to be 29 million people [19], 81% of whom were living in rural areas in 2015 [20]. Nepal ranked at the poorest end of the United Nations Development Programme Human Development Index in 2016, in the 144th position out of 188 countries [21]. Water scarcity is a common issue in the country [22,23] that is exacerbated by ongoing climate change impacts [24]. Developmental efforts in the past years have mainly focused on meeting the water supply demand and increasing freshwater accessibility. In addition, recent national development initiatives have focused on eliminating open defecation and achieving universal improved drinking water access, especially in rural areas [25]. The Nepal Water Supply, Sanitation, and Hygiene Sector Development Plan for 2016–2030 [26] highlights poor drinking water quality and the lack of an effective monitoring and surveillance system as a barrier to the implementation of the National Drinking Water Quality Standards [27].

**Figure 1:**
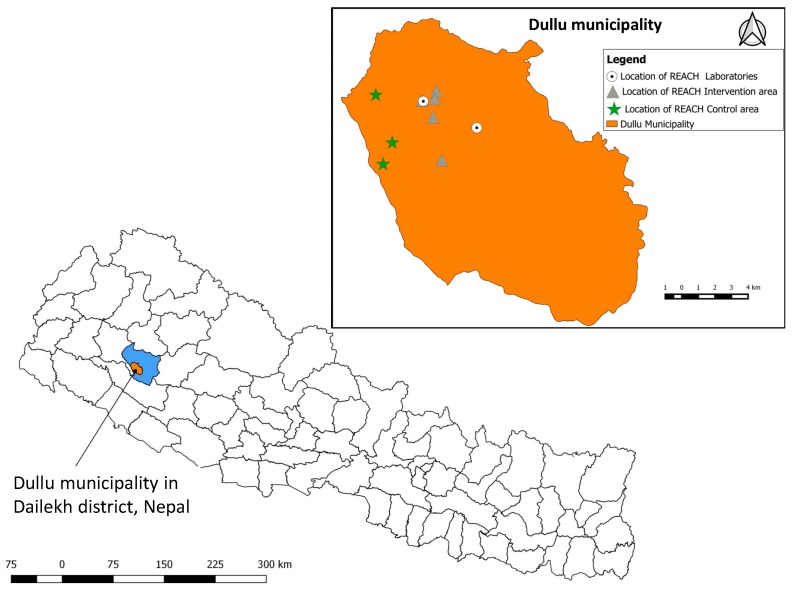
Map of Nepal with the district borders highlighting Dailekh district in blue and the intervention area, Dullu municipality, in orange. The map inset expands on the intervention area, showing the locations of the intervention and control schemes in relation to the field laboratories.

The study was conducted in the Dullu municipality in the Dailekh district of the Mid-Western Development Region (Figure 1). This intermediate hilly region was selected as the study location because it is representative of the rural, hilly settings of Nepal, with the additional advantages of close proximity to sufficient projects within the Helvetas-Nepal IWRM service area and relatively convenient road access. In total, eight communities with gravity-fed piped drinking water schemes were selected for this study: five schemes where risk-based water safety intervention took place (hereafter called intervention schemes) and three control schemes where no risk-based water safety interventions were implemented.

Before the study, all eight communities had received a new piped water system with private or public taps constructed by Helvetas-Nepal between 2012 and 2016. Alongside system installation in each community, the program additionally established a water and sanitation users’ committee, promoted improved household hygiene practices, distributed ceramic filters for household water treatment, and trained a female community health volunteer and a village maintenance worker responsible for repairing the water supply system. These pre-baseline activities, which defined the starting scenario of all the study communities, are summarized in Table 1.

### 2.2. Description of Drinking Water Schemes

**Figure 2:**
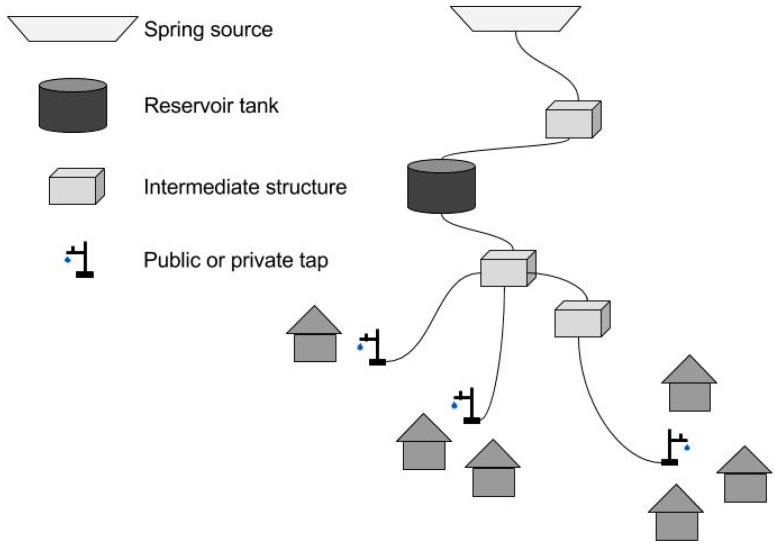
Sketch of a typical gravity-fed piped water scheme (or sub-scheme). Each scheme is composed of 1–4 sub-schemes. Sub-schemes comprise one water project for the same community but make use of independent water sources. Within a sub-scheme, one water source can feed several reservoir tanks that distribute water to different areas of the village. The intermediate structures can be distribution and collection chambers, purge valve chambers, break pressure tanks or interruption chambers, and air valve chambers.

The selected water schemes were constructed between 2012 and 2016; all were completed at least one year prior to this research. All are simple gravity-fed piped networks with spring sources, except one that includes a solar-powered lifting pump to deliver water from a downhill reservoir to the uphill distribution tanks. All the schemes provide intermittent water services with variable opening times and service durations throughout the year, as is common in the hilly region. They are all similar in their layout with a spring source that is connected to a reservoir tank by a distribution line, with water then flowing to the taps (Figure 2). All the selected schemes deliver water to public taps except one that has private taps only.

### 2.3. Study Design and Sample Strategy

Two distinct research strategies were used: one for the baseline and endline surveys and the other for regular monitoring. The baseline and endline surveys aimed to assess community members’ perceptions and behaviors regarding their drinking water. The sanitary state of the water schemes and the microbial water quality were also assessed at the baseline and the endline to measure changes before and after the water safety intervention. By contrast, regular monthly monitoring activities served as less intensive “spot checks” to capture temporal variations in water quality and sanitary indicators. In this way, regular monitoring data informed the ongoing implementation of interventions within each scheme by gauging their effectiveness and identifying any unaddressed system vulnerabilities.

#### 2.3.1. Baseline and Endline Surveys

The baseline data collection took place in June 2017 and the endline data collection in January 2018. The field teams were composed of staff members of Eawag, Helvetas-Nepal, and the local non-governmental organization (NGO) Social Services Center. All the questionnaires were translated and conducted in Nepali. Only households using the water scheme were eligible for enrollment. Eligible households were selected randomly from the water project beneficiaries list and enrolled following informed consent about the project’s purpose and anonymity of the questionnaire. At the study baseline, if the household declined to participate in the study or if no adult was available at the time of the visit, another household was selected randomly as a replacement. A total of 15 households were enrolled at each water scheme for a total of 120 surveys. During the endline period, the same households from the baseline were interviewed. The survey questions probed the households’ drinking water supply characteristics, sanitation and hygiene practices, and socio-economic statuses. A drinking water sample was taken at each household by collecting 100 mL of water in the same manner as if getting a cup of water to drink. At each of the 8 study schemes, water samples were also taken at the inlet of all reservoir tanks and from three randomly selected taps during the baseline and endline visits.

#### 2.3.2. Regular Monitoring

At each of the five intervention schemes, one source, one reservoir tank, one tap, and one household were regularly monitored every three–six weeks between August and December 2017 for both drinking water quality and sanitary status (Table 2). Sanitary inspection forms for sources, reservoir tanks, taps, and stored water were developed based on the updated forms provided by the WHO [17], with modifications made to suit the field context. Each form was composed of 10 yes/no questions from which a risk score out of 10 points was calculated, with a higher risk score indicating a greater health risk posed at the specific point (see Appendix Table A1 for the content of each sanitary inspection form). A trained person from each WSP task force was responsible for selecting monitoring points, taking the water samples, and performing the sanitary inspections. Monitoring points were rotated each month and were all water-connected: the household used water from the corresponding tap that was connected to the reservoir tank and the source that was being monitored. Care was taken to ensure that households were not aware of monitoring visits in advance. Regular monitoring is planned to be continued after the study’s end as an integral part of the water safety framework. Further details on the regular monitoring strategy are provided in Supplementary Materials Section S1.

### 2.4. Water Safety Plan, Interventions, and Laboratories

A WSP approach was adopted within the intervention communities. A WSP task force was formed as a subgroup of the pre-existing water users’ committee. The task force members’ main responsibilities were to evaluate and identify risks to their water scheme and to support efforts towards improved water security management practices. Based on the full sanitary inspection performed at the baseline, the WSP task force and Helvetas-Nepal’s technical team collaboratively decided on one or more scheme upgrades to improve the water quality and devised a participatory approach for implementation.

The five intervention schemes received the system upgrade measures shown in Table 1 during November and December 2017. Additional details on the water scheme upgrades are provided in the Supplementary Materials Section S2. The upgrading process was based on a participatory approach that emphasized community members’ involvement in the decision making to increase their sense of ownership over the project [28]. The communities also contributed to the system upgrade efforts by providing unskilled labor and local materials for construction.

In addition, two water quality laboratories were installed at a village health post and a secondary school close to the five interventions schemes. These laboratories consisted of a simple field incubator connected to a solar photovoltaic setup and all the materials required to perform the microbial water quality analysis (*E. coli* and total coliforms). The laboratory technicians received targeted group training followed by supervised field work. All the data gathered during the regular monitoring was collected by the trained WSP task force members and lab technicians under the supervision of a local NGO staff member who had also previously received intensive training.

### 2.5. Data Collection Tools and Water Quality Analysis

#### 2.5.1. Mobile Data Collection

All the data, including the baseline and endline household surveys and regular sanitary inspections, were collected using tablets (Samsung Galaxy Tab A, Seoul, Korea) equipped with the Akvo Flow application (Akvo Foundation, Amsterdam, The Netherlands). The data were uploaded to the cloud and made available to project team members to be analyzed remotely.

#### 2.5.2. Water Sampling and Microbial Water Quality Testing Protocol

Water samples collected at the reservoir tanks were taken directly from the inlet, which is the closest point to the water source that was available to sample; therefore, the sample collected is representative of the water entering but not the water being stored at the reservoir tank. At the taps, water was run for 30 s before sampling to wash out any deposited residue and ensure a representative sample from the piped system. The household water samples were collected at the point of consumption (i.e., 100 mL of water was collected in the same way a glass of water for drinking would be prepared). All the water samples from a single scheme were collected on the same day. The water samples were collected in sterile 100 mL Whirl-Pak sampling bags (Nasco, Fort Atkinson, USA). For chlorinated schemes, Whirl-Pak Thio-bags (Nasco, Fort Atkinson, USA) containing sodium thiosulfate were used to inactivate any residual chlorine. Because the electricity required to support a cold chain was not available, the samples were transported to the field laboratories in cooler boxes without ice. The samples were processed by membrane filtration using Nissui Compact Dry EC plates (Nissui Pharmaceuticals, Tokyo, Japan) and a modified filtration device (DelAgua, UK), followed by incubation at 35 ± 2 °C for 24 h. All the samples were transported and processed within two hours of collection. If transportation to laboratories within two hours was impossible, the samples were processed on site and incubated later. A detailed protocol for the membrane filtration method and further information on the construction of the field incubators are available in the Supplementary Materials Sections S3 and S4, respectively.

#### 2.5.3. Bacteria Enumeration and Quality Control

After incubation, *E. coli* and total coliform were enumerated on Compact Dry EC according to the manufacturer’s instructions. Counts higher than 300 colonies per plate were reported as too numerous to count (TNTC). The results are reported as colony forming units (CFU) of total coliforms or *E. coli* per 100 mL (CFU/100 mL). To assess the replicability of the method, a duplicate was performed every tenth sample during the baseline and endline data collection. In addition, a random duplicate was taken from one of the sampled sites (tank, tap, or household) during each round of a scheme’s regular monitoring. Negative controls (blanks) were processed daily. The statistical analyses of all control measures are found in the Supplementary Materials Section S5.

### 2.6. Data Analysis

Water quality and survey data were initially compiled and cleaned using Excel 10 (Microsoft, Redmond, WA, USA). Coding and statistical tests of intervention effects were performed using IBM SPSS (IBM, New York, NY, USA). The microbial concentrations were observed to be exponentially distributed; therefore, bivariate comparisons made use of non-parametric tests (e.g., Mann–Whitney U test and central tendency reported as median CFU/100 mL) or parametric tests (e.g., Student’s *t*-test for independent samples following Log_10_ transformation of *E. coli* data and central tendency reported as mean CFU/100 mL). For all Log_10_ transformations, zero counts were set to 0.5 CFU/100 mL and TNTC values were set to the upper limit of detection (300 CFU/100 mL).

### 2.7. Ethics Statement

All participating households gave their informed consent before being interviewed. The research was conducted in accordance with the Declaration of Helsinki, and the protocol was approved by the Eawag ethics committee (protocol 16_09_072017). The study received government approval in Nepal as part of the Helvetas-Nepal IWRM research program.

## 3. Results

### 3.1. Household and Drinking Water Scheme Characteristics

#### 3.1.1. Generalities

The average household had 6.5 (SD = 2.3) family members, with 0.8 children who were 5 years old or younger. Virtually all of the interviewed households (99%) were active in agricultural and farming activities. The monthly expenses per household ranged from 1550 to 50,000 Nepalese Rupees (NPR, M = 10,610, SD = 7800), corresponding to 15.5–500 United States Dollars (USD, M = 106, SD = 78) using a rounded average currency exchange rate of 2017 (Exchange rate calculator, http://www.x-rates.com/average/?from=USD&to=NPR&amount=1&year=2017). When asked about their main concern within their community in the baseline survey, households most frequently mentioned water supply services (31% of intervention and 53% of control households). Most of the households interviewed had walls made of wood or mud (>73%), a floor made of mud, sand, or dirt (>87%), and a roof made of metal (>36%) or thatch (>14%). Concerning sanitation, most of the households reported using an improved private latrine (>89%). There was no electrical grid in the project area, but most households had installed small private solar systems to power lighting and mobile phones (>89%). A further description of the household characteristics is available in the Supplementary Materials Section S6.

#### 3.1.2. Hygiene Practices and Reported Illness

At the baseline visit, most households reported washing their hands after going to the toilet (>91%), before eating (>93%), and before cooking (>67%). The frequency of soap use during handwashing increased from 43% to 63% between the baseline and endline among the households using the intervention schemes, whereas the frequency decreased from 80% to 60% among the control schemes over the same period. The availability of dedicated handwashing stations with a faucet increased at the intervention schemes from 65% to 83% and stayed constant at the control schemes at 82%. The Supplementary Materials Section S6 contains detailed results of the households’ handwashing practices.

Most households (96%) did not report having experienced any diarrhea or respiratory illness cases among their family members in the week prior to the survey. A total of six people at the baseline and four people at the endline had experienced a case of diarrhea or respiratory illness, with about half of these cases being children under the age of five. All the households reporting illness during the baseline were using the interventions schemes, whereas at the endline most of the households reporting illnesses (three of the four) were using a control scheme.

#### 3.1.3. Perception of Drinking Water Quality and Water Treatment Practices

**Figure 3:**
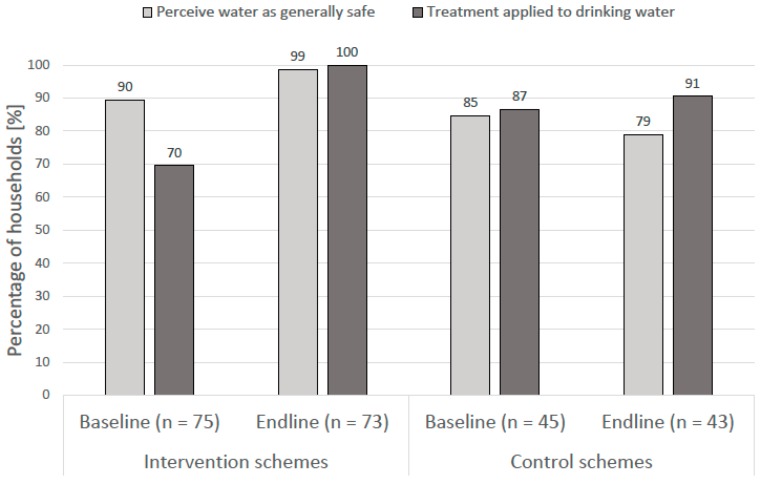
Drinking water safety perception and treatment coverage among the households served by the intervention and the control schemes from the baseline to the endline period.

At the baseline visit, most households perceived their drinking water taste and smell as good (>98%), color as clear/good (>92%), and as generally safe to drink (>85%). By the endline visit, the share of households reporting their drinking water was safe had increased slightly in the intervention schemes (99%) and decreased in the control schemes (79%) (Figure 3). However, households using the interventions schemes that received chlorination as part of the WSP intervention reported greater dissatisfaction with the taste of the water by the endline visit; among the two schemes where chlorination was introduced, chlorine taste and “bad or funny smelling water” was reported by 15% and 14%, respectively, of the 29 households interviewed. Further details on the perceptions of the drinking water quality are available in the Supplementary Materials Section S6.

Regarding water treatment practices, at the baseline visit, fewer households in the intervention schemes reported treating their drinking water (70%) compared with the households in the control schemes (85%). The share of the households adopting the treatment practices increased among all households from the baseline to endline visits (Figure 3). However, the observed difference in the treatment coverage was only statistically significant among the intervention schemes (c^2^ (1, *n* = 147) = 26.18, *p* = 0.00), with all the households reporting that they practiced some form of household water treatment by the endline.

At the baseline, the households that said their drinking water was not generally safe indicated the main reasons as being an unprotected source (36%) or animal waste (29%). However, more than a quarter (29%) of the households did not know why they thought the water was unsafe. At the endline, half of the households that did not consider their water to be safe reported toilet waste as the major reason. The other major concerns mentioned at the endline included animal waste (38%), an unprotected source (25%), and chemicals (13%).

#### 3.1.4. Water Supply Characteristics

In Nepal, efforts have been made in recent decades to provide access to an improved drinking water supply for all rural households. In the study area, the designs of the gravity-fed schemes are all similar, with source water directed to one or several reservoir tanks, which are then opened daily for distribution. The study schemes served from 29 to 108 households or 177–683 people (see the additional scheme characteristics in Table 3). The water services were intermittent, meaning that reservoir tanks were manually opened once or twice per day at a defined hour. The opening times and durations varied throughout the year depending on the source water availability and the time required to fill the reservoir. Usually, the opening duration ranged from one to two hours, with shorter times during the dry season.

All the water points within the study communities were functional at the time of the research team’s baseline and endline visits. Most households (>80%) reported that their water supply scheme functioned well in general, and most (>85%) reported that they were confident that their water system would still be functional in a year. Most of the interviewed households (>82%) had access to a public tap, and among these households, nearly all (>95%) reported it as their main drinking water source. The average reported time taken for a round trip to the drinking water source, including queuing time, was 10 min (SD = 9). A trained local maintenance worker was responsible for regular maintenance and repairs for each scheme. Most interviewed households (>87%) reported that they could get help from their local maintenance worker for necessary repairs and that repairs could be completed within a week (>71%). A water tariff system had been implemented prior to the start of this study, with most households (>85%) reporting that user fees were collected to pay for repairs on an as-needed basis. Detailed water supply characteristics are available in the Supplementary Materials Section S6.

#### 3.1.5. Water Supply Management

The household survey probed the community members involved in the management, operation, and maintenance of their water supply scheme. A total of 44% of the households interviewed at the endline indicated having a family member who was either a member of the water and sanitation users’ committee or the WSP task force or had served as a maintenance worker, community health volunteer, or tap stand care taker. The water users’ committee met together regularly (most often monthly) to discuss issues related to the water supply scheme. During the construction of the scheme, the water users’ committee also met with community members monthly to discuss the project, establish a fund for operation and maintenance, assign maintenance workers, collect contributions toward construction, and eventually, conduct public reviews of the committee’s income and expenditures. After construction was completed, the water users’ committee generally met with community members only once every year or every second year to perform the aforementioned duties, as well as reform the water users’ committee as needed. During the baseline, 60% and 40% of households using the intervention and control schemes, respectively, indicated that they were aware of the water users’ committee meetings within their community. At the endline, these percentages increased to 79% and 67% for the intervention and control schemes, respectively, suggesting that the study served to raise awareness regarding the water users’ committee activities. More detailed results are available in the Supplementary Materials Section S6.

#### 3.1.6. Activities within Intervention Schemes

Among the households served by the intervention schemes only, additional questions were asked at the endline visit to assess the activities taking place during the WSP implementation. Nearly all (88%) the households served by the intervention schemes were aware of the WSP strategy, and among these households, about half (54%) had participated in its development and implementation through their membership in the WSP task force, involvement in the regular scheme chlorination, or the installation of the intake filter. A total of 93% of households had heard about the laboratories that had been installed for monthly water quality testing, and 71% said that the results of the microbial analysis had been reported back to them by local NGO staff members or members of the water users’ committee. Among the 51 households that had received their test results, 37% indicated that their water quality was contaminated. In response, all of these households had begun to treat their water using a ceramic candle filter (100%) and boiling (16%). When asked about their desire for future water quality testing, 96% of interviewees responded positively and said they would pay up to 500 NPR (or 4.78 USD) per test, with a median value of 50 NRP (or 0.48 USD) per test (Exchange rate calculator, http://www.x-rates.com/average/?from=USD&to=NPR&amount=1&year=2017).

Among the nine households served by intervention schemes that had a family member in the water users’ committee, all had been informed about the results of the monthly water quality monitoring. All but one of the households had then discussed these results with the water users’ committee, and in about half of the instances (44%), actions to improve the water scheme had been undertaken. Further details on the activities within the intervention schemes are provided in the Supplementary Materials Section S6.

### 3.2. Water Quality Analysis

#### 3.2.1. Household Stored Water Sample Characteristics

Among all the households, most of the water samples collected from the stored water containers were clear at both the baseline (>96%) and the endline (>81%) visits. The share of stored water samples treated by household ceramic filters or boiling among the intervention schemes increased from 63% at the baseline to 100% at the endline (Table 4). By the endline visit, three-quarters of these samples had also received some form of scheme-level treatment, such as chlorination; however, no monitoring of chlorine residual in the stored water was conducted as confirmation. By contrast, the share of stored water samples that had been treated at the household level within control schemes remained relatively constant, from 76% at the baseline to 86% at the endline (see the Supplementary Materials Section S6 for additional details).

#### 3.2.2. Baseline Water Quality and Qualitative Sanitary Observations

At the study baseline, the microbial water quality was assessed at each of the surveyed households, as well as at the all the reservoir tanks and three taps per scheme. All the data were analyzed based on *E. coli* concentrations unless otherwise stated. Table 5 shows the median and the mean Log_10_ *E. coli* contamination at the intervention and control schemes. The Mann–Whitney U tests showed no statistical differences in the *E. coli* concentrations between sampling points at the intervention and controls schemes at the baseline (*p* ≥ 0.05).

The sanitary inspections of the water schemes at the baseline visit indicated high risk scores at all the spring sources due to inadequate protection measures. The infiltration of contaminated runoff water and open intakes were the main hazards identified. Additionally, for most of the spring sources, the inspections revealed that intake maintenance was not possible without compromising the integrity of the intake covering and the protective gravel and sand layers. Any blockage at the intake would require the removal of these covering layers, thereby risking that the intake would not be properly covered afterwards. Generally, the other structures, such as the reservoir tanks and the distribution pipes, were in good condition. Nevertheless, the tank covers were pinpointed as vulnerabilities, because contamination could enter during rain events or when the covers were opened. Occasional pipe leaks were observed, and the taps were found to be damaged or leaking in some of the schemes.

#### 3.2.3. Monthly Monitoring of Intervention Schemes

**Figure 4:**
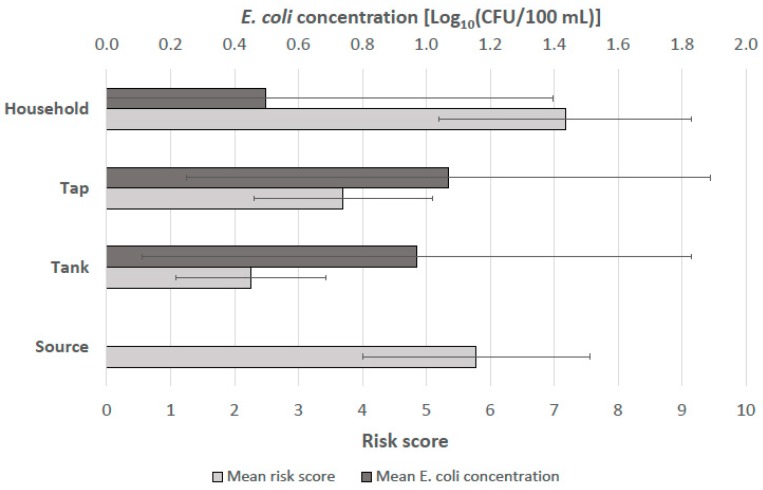
Mean risk scores from monthly sanitary inspections and mean *Escherichia coli* concentrations from monthly sampling (*n* = 23, standard deviation bars shown). The maximum risk score is 10.

Regular monitoring of the intervention schemes included water quality testing and structured sanitary inspections that provided a calculated risk score. Figure 4 shows the mean risk scores and mean *E. coli* concentrations at the source, reservoir tank, tap, and household. The microbial water quality was not measured at the sources because no samples could be collected without damaging the integrity of their protective structures.

The average risk score was higher at the sources (due to poor protective measures) and the households (due to recontamination vulnerabilities) than at the taps and reservoir tanks. However, the microbial water quality of household stored water was on average better than at the taps and reservoir tanks. With these results, the sanitary inspections did not accurately predict the water quality test results at each given point. The household water treatment practices appeared to improve the stored water quality, even if the overall sanitary state of the household was poor according to the inspection forms. Rain events during the monitoring day and the preceding day were recorded in the sanitary inspection forms and examined as a potential factor explaining variations in the microbial water quality. However, the results did not reveal any meaningful impact of rain on the observed microbial concentrations.

#### 3.2.4. Endline Water Quality and Qualitative Sanitary Observations

Water quality at the endline was assessed at the same points as during the baseline (Table 5). The Mann–Whitney U tests showed a small but significant difference in the *E. coli* contamination levels of the household stored water samples between the intervention (median = 0 CFU/100 mL) and control schemes (median = 4 CFU/100 mL), U = 1073, *p* = 0.004. No significant differences in the *E. coli* contamination of the intervention and control scheme reservoir tanks or taps were observed (*p* ≥ 0.05).

The sanitary inspections during the endline visit showed that all the source intakes of the intervention schemes had been structurally improved. Each had a new intake filter made of fine sand and gravel layered and packed in a net. The intake was also topped with a plastic cover to avoid surface water infiltration. Rain water diversion ditches were constructed around the source intakes to prevent rainwater runoff from entering the intake area. In some cases, additional shields against landslides were installed as added protection. Protection and regeneration of the micro-catchment through the 3R (Recharge, Retention, Reuse) intervention (see Table 1) were observed but only at their early stages. It is expected that this plantation work will deliver its full potential as a conservation measure several years after its completion. The intervention schemes were also improved through the replacement or repair of leaking pipes throughout the network and improved maintenance of the public taps.

### 3.3. Comparisons of Fecal Contamination at the Baseline and Endline Measurement

#### 3.3.1. Average Contamination by Scheme and Sampling Point

**Figure 5:**
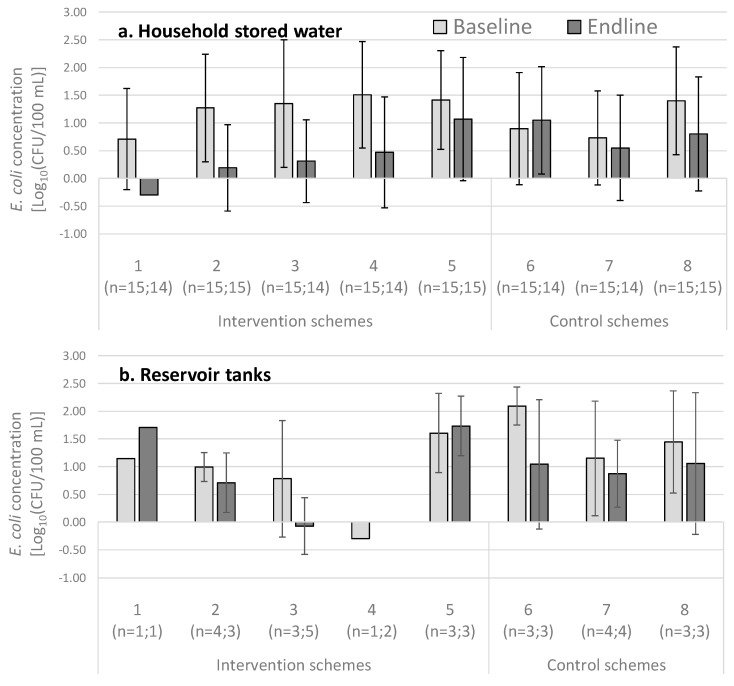

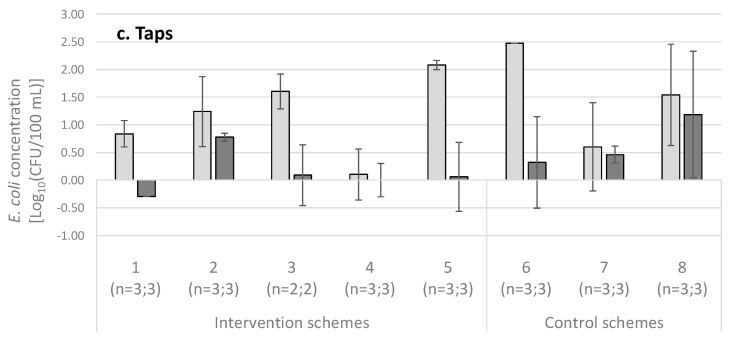
Mean *E. coli* concentrations of the (**a**) household stored water containers, (**b**) reservoir tanks, and (**c**) taps at the baseline and endline for each of the eight schemes. The standard deviation bars are shown.

The mean *E. coli* contamination of household stored water is shown in Figure 5a. These results showed that the contamination during the baseline was on average greater in the intervention schemes than in the control schemes. By the endline visit, the opposite situation was observed, with most intervention schemes having lower contamination levels within the stored water on average, as compared with the control schemes. At most reservoir tanks and taps at the intervention and control schemes, the water quality at the baseline had improved by the endline visit (Figure 5b,c). A particularly high level of fecal contamination was observed in the reservoir tanks and taps of the control scheme number six during the baseline visit.

**Figure 6:**
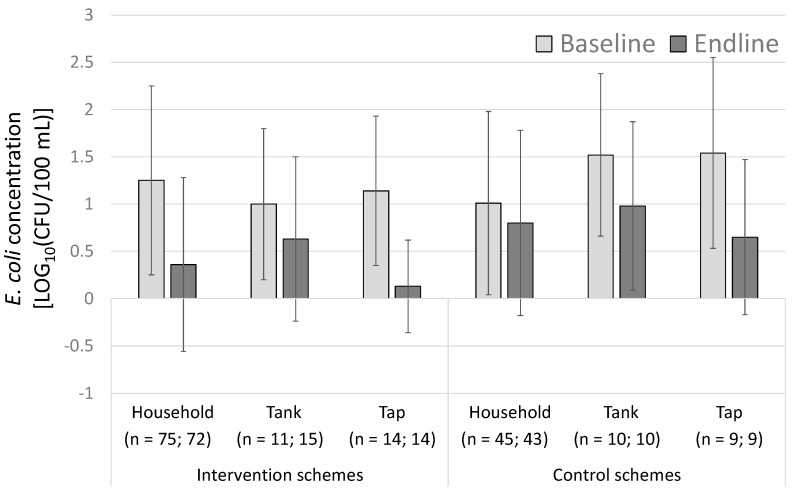
Mean *E. coli* contamination at each sampling point at the baseline and endline measurements for the intervention and control schemes. The standard deviation bars are shown.

Figure 6 shows the mean *E. coli* concentrations for the intervention and control schemes at each sampling location. The greatest reductions in the contamination between the baseline and endline measurements are seen at the households and taps among the interventions schemes (see the Supplementary Materials for additional microbial analyses across the sampling points (Section S7); within the chlorinated schemes specifically (Section S8); among the households using and not using ceramic water filters (Section S9); and other detailed microbial results (Section S10)).

#### 3.3.2. Statistical Comparisons of Fecal Contamination at the Baseline and Endline Measurements for Intervention and Control Schemes

A Student’s *t*-test was used to compare the *E. coli* contamination levels at the baseline to the endline measurements within the intervention and control schemes (Table 5). The results show a statistically significant difference in mean contamination levels at the households and the taps within the intervention schemes only. The mean Log_10_ *E. coli* concentration at the households served by the intervention schemes was 1.25 CFU/100 mL at the baseline and 0.36 CFU/100 mL at the endline. At the intervention scheme taps, a reduction in the mean Log_10_ *E. coli* concentration from 1.14 CFU/100 mL to 0.13 CFU/100 mL was observed. No significant difference in the average contamination levels between the baseline and the endline was observed at the intervention reservoir tanks or at any of the sampling points in the control schemes (see the Supplementary Materials Section S10 for further discussion and statistical analysis).

When examining whether the samples met the WHO guidelines for drinking water safety (<1 CFU *E. coli*/100 mL), the results show that the share of the household stored water samples from the intervention schemes with no detectable *E. coli* increased significantly from 17% at the baseline to 53% at the endline (c^2^ (1, *n* = 147) = 24.01, *p* = 0.00). Also significant was the increase in the tap samples from the intervention schemes that met the WHO guidelines, from 7% at the baseline to 50% at the endline (c^2^ (1, *n* = 28) = 6.30, *p* = 0.03), with all the tap samples at the endline having less than 10 CFU *E. coli*/100 mL. Other sampling points did not yield meaningful changes in the share of samples meeting the WHO criteria (see Appendix Table A2 for detailed results and the Supplementary Materials Section S11 for temporal representations of the baseline, endline, and regular monitoring data).

#### 3.3.3. Difference-in-Differences Analysis

A difference-in-differences analysis was used to compare the household water quality data from the intervention and control schemes at the baseline and the endline. Estimating the natural change at the control sites and subtracting it from the intervention sites indicated that the effect of the interventions on the household water quality caused a decrease of the mean Log_10_ concentration of *E. coli* of 0.681 CFU/100 mL (SE = 0.26, *n* = 235, *t* = −2.614, *p* = 0.01) among the intervention schemes.

The difference-in-differences analysis for the water quality at the reservoir tanks was +0.168 Log_10_ CFU/100 mL and at the taps was −0.13 Log_10_ CFU/100 mL. This is interpreted as meaning that the interventions were responsible for an increase in the contamination at the reservoir tanks and a decrease at the taps (reservoir tanks: DD = 0.168, SE = 0.512, *t* = 0.329, *p* = 0.744, *n* = 46; taps: DD = −0.13, SE = 0.464, *t* = −0.281, *p* = 0.78, *n* = 46). This unexpected finding could be explained by the fact that the control scheme number six showed exceptionally high contamination at the baseline as compared with all the other schemes (Figure 5b), resulting in a large improvement of the mean water quality at the control schemes’ reservoir tanks. The difference-in-differences analysis at the taps is aligned with the results presented above and indicates a statistically significant improvement in the water quality due to the interventions.

## 4. Discussion

### 4.1. Study Novelty and Insights

While past studies have investigated water safety interventions in rural areas of Nepal, to the authors’ knowledge, no study to date has reported outcomes based on comparison to a set of control communities. The aims of this research were to describe an approach for improving the drinking water safety that is adapted to this unique setting, as well as to rigorously evaluate whether this strategy was capable of achieving measurable improvements in the water quality. The findings reported here will be of interest to government agencies, water program managers, system operators, and program managers throughout Nepal and are applicable to other remote rural areas dependent on gravity-fed piped supplies.

This study revealed several insights relevant to the rural water sector. First, we observed universal uptake of the household water treatment (ceramic water filters and/or boiling) within the intervention communities. This finding suggests that the suite of water safety interventions delivered through the WSP, including the intensive WASH promotion activities, were very effective in motivating behavior change over an eight-month period. The WASH promotion activities included the communication of the stored water quality results to most households following testing. The survey data revealed that all the households who received the results indicating contamination of their stored water subsequently adopted treatment practices. Moreover, nearly all the survey respondents said that they would be interested in further water quality testing at an average price of 0.70 USD per test.

The high uptake of the household water treatment and increased demand for water quality testing among the households could also be attributed to a generally high level of awareness and involvement among the community members. For example, 88% of survey respondents said they knew about the WSP activities within their community. Over half of these households had served as an official member of the WSP task force or participated in infrastructural improvements, such as the installation of intake filters or the implementation of chlorine dosing. Taken together, these results suggest the broad level of engagement by the households in the planning and implementation of their water safety interventions contributed to the successful outcome observed over an eight-month period. More generally, the study findings suggest a dynamic interaction between the community members’ participation in the water supply stewardship, the delivery of targeted water quality information, and the demand for safe drinking water.

A second insight from this research is that the sanitary inspection risk scores did not accurately predict the microbial water quality at different points across the system. According to the sanitary inspection metric used, the risks were on average greatest at the sources and households and lowest at the reservoir tanks and taps. These findings were driven by the poor physical protection of the sources and factors indicating the recontamination potential of the stored drinking water at the household level. Surprisingly, however, the water quality measurements revealed the opposite trend; the fecal contamination of the household stored water was on average lower than at the collection taps and reservoir tanks (it was not possible to measure the microbial water quality at the source). These findings may be explained by the uptake and consistent use of ceramic water filters by the households following enrollment in the study, thereby improving the water quality even if other measures of the household’s sanitary state remained poor according to the inspection form.

Finally, statistical comparisons of the microbial water quality revealed improvements at all points monitored for both the intervention and control schemes. However, the improvements observed in the average *E. coli* concentrations from the baseline to the endline were only statistically significant for the taps and the household stored water containers in the intervention group (and not so in the control group). Examining the microbial data at the scheme level, we found that the household stored water quality consistently improved from the baseline to the endline for all the intervention schemes, whereas an inconsistent trend was observed for the three control schemes. In addition, the intervention communities showed universal adoption of household water treatments by the endline, resulting in over half of the households having stored water meeting the WHO guidelines for water safety (0 CFU *E. coli*/100 mL). The reduction in the fecal contamination among the intervention taps is notable as well, with half of the taps meeting the WHO guidelines (up from only 7% at the baseline) and all the taps delivering water with less than 10 CFU *E. coli*/100 mL. These results, while promising, do not indicate perfect compliance with international water safety guidelines for all the intervention schemes. Thus, the water safety interventions applied may be considered as an effective and viable interim solution in efforts to eventually achieve universal access to safely managed drinking water in rural settings.

### 4.2. Study Limitations

Some features of this study design limit our ability to generalize the findings beyond the sampled population. Most notable is the generally high level of water service experienced within both the intervention and control schemes. Nine out of every 10 survey respondents reported at the baseline that their main water source tasted and smelled good and was generally safe to drink. Moreover, all the water points were functioning at the time of the research team’s visit, and most survey respondents believed it would likely continue to function well over the coming year. This generally high level of satisfaction and confidence among water users may be unique to the program setting and is likely a driving factor in the households’ willingness to pay for water services and engage in stewardship of the infrastructure over time.

Another issue is the enrollment of only three control schemes as compared with the five intervention schemes. This research design limitation was driven by both resource constraints and ethical considerations; within a set budget, there was the need to ensure the potential benefits of the study (water supply upgrades) outweighed the potential costs (lost time due to participation). As a result, the sample sizes in the control group were roughly three-fifths the size of those in the intervention group, which underpowered comparisons of small effective sizes. Finally, the follow-up period for this study was eight months, which only allows for preliminary conclusions to be made regarding the sustainability of the interventions examined. Future research should ideally monitor the outcomes reported here over a longer period (at least one year and ideally up to five years). This is especially critical for understanding the sustainability of behavioral measures known to decline over time, such as household ceramic filter use.

### 4.3. Recommendations for Water Sector Policy and Practice

There are several recommendations for water sector practitioners arising from this research. First, these study results indicate that over the short term (eight months), the applied water safety interventions were highly effective in motivating uptake and use of household water treatments. Such promotional activities were tailored to the needs of the households in rural Nepal and were integrated into a broader WSP framework. To replicate this success, program managers should strive for a comprehensive approach that merges household-centered WASH promotional activities with system-scale water safety efforts. Second, sanitary inspection scores did not reliably predict the microbial concentrations at various sampling points and are therefore insufficient for assessing actual health risks due to drinking water consumption. Based on these findings, standardized sanitary inspection packages should be combined with regular water quality testing for a comprehensive risk management approach. Finally, the applied interventions, while effectively improving water quality at the taps and in the household stored water containers, did not achieve perfect compliance with the international guidelines over the eight-month study period, in part, because interventions such as micro-catchment restoration required more time to deliver their intended benefits. Future research should therefore explore additional treatment options, for example, disinfection by automated chlorine dosing or ultraviolet treatment devices. 

## 5. Conclusions

This study characterized and assessed a risk-based strategy for improving the drinking water quality of gravity-fed piped schemes in the hilly regions of Mid-Western Nepal. This research was motivated by the need to accelerate progress towards achieving universal access to safely managed drinking water in similar contexts, where effective treatment and regular monitoring of piped supplies is often challenged by geography, limited resources, and unreliable supply chains. The results showed that simplified field laboratories equipped for microbial testing can inform ongoing decision-making regarding targeted system upgrades and mitigation measures. These interventions led to positive changes in the drinking water quality at the taps and within the households over eight months of implementation. Of particular note was the achievement of 100% coverage of household water treatments with ceramic filters and boiling across all intervention schemes. In addition, the results showed high levels of involvement by the households in planning and implementing the WSP within their community, especially through regular engagement with the local water and sanitation users’ committee.

The study also revealed the inconsistent predictability of microbial contamination using standard sanitary inspection forms alone. This finding suggests that such forms, while useful for identifying potential hazards, should be combined with regular water quality testing for a comprehensive risk management approach for piped schemes. By the study endline visit, half of the samples collected from households’ stored water containers and taps were free of fecal contamination—a significant improvement from the baseline visit when only 17% and 7% of the households and the taps, respectively, met the international guidelines for microbial safety. Despite all the water points sampled not meeting the stated guidelines, the applied strategy nevertheless proved promising as an intermediate step towards achieving universal access to safe drinking water in rural areas.

## Figures and Tables

**Table 1:** Activities carried out before and during the study period within intervention and control communities.

Activity	Intervention Communities	Control Communities
Helvetas-Nepal program activities established before the study	Constructed piped water scheme	Same as intervention schemes
	Established water users’ committee	
	Conducted household hygiene campaign	
	Installed ceramic water filters	
	Trained community health volunteer and village maintenance worker	
Data collection at study baseline and endline	Household survey	Same as intervention schemes
	Water quality sampling	
	System sanitary inspection	
Physical upgrades to water schemes	Source protection	None
	Intake improvement	
	Scheme level chlorination ^1^	
	Small repairs	
	3R measures (Recharge, Retention, Reuse)	
Management interventions	Creation of the Water Safety Plan task force	None
	Regular monitoring of sanitary state and water quality	
	Laboratory coverage	
	Improved maintenance	
Behavior change interventions	Promotion of good handling practices for ceramic candle filter	None
	Household sanitary inspections	

^1^ Two of the five intervention schemes received chlorination.

**Table 2:** Quantity of water samples at each phase of sampling.

Sampling Phase	Household	Tank	Tap
Baseline	120	21	23
Regular monitoring	23	23	23
Endline	115	25	23

**Table 3:** Description of water supply schemes characteristics: mean (standard deviation), [range].

Characteristics	Intervention Schemes	Control Schemes
Households served	66.8 (32.2), [29 to 108]	84.3 (30.4), [50 to 108]
Population served	411.8 (209.5), [177 to 683]	511.7 (194.9), [292 to 664]
Spring sources	2.6 (1.1), [1 to 4]	3.3 (1.2), [2 to 4]
Reservoir tanks	3.2 (1.5), [1 to 5]	3.7 (0.6), [3 to 4]
Taps	19.4 (3.6), [15 to 24]	26.7 (14.2), [18 to 43]

**Table 4:** Characteristics of stored water samples collected from households.

Sample Characteristic	Intervention Schemes		Control Schemes	
	BL (%)	EL (%)	BL (%)	EL (%)
Sample collected from:				
Ceramic candle filter outlet	57	99	78	81
Gagri/jerrycan/bucket	43	1	22	19
Visual quality:				
Clear	100	97	96	81
Somewhat turbid	0	3%	4	19
Very turbid	0	0	0	0
Received treatment at:				
Household level only	59	25	76	86
Scheme level only	0	0	0	0
Both household and scheme level	3	75	6	0
No treatment	37	0	18	14

BL: Baseline; EL: Endline.

**Table 5:** *Escherichia coli* concentrations at each sample location for the intervention and control schemes, with bivariate comparisons of the mean *E. coli* contamination at the baseline and endline measurements.

Location	Sampling Phase	Intervention Schemes				Control Schemes			
		*n*	Median [CFU/100 mL]	Mean (SD), [Range] [Log_10_(CFU/100 mL)]	Student’s *t*-test	*n*	Median [CFU/100 mL]	Mean (SD), [Range] [Log_10_(CFU/100 mL)]	Student’s *t*-Test
**Household**	Baseline	75	24	1.25 (1.00), [−0.30 to 2.48]	*t* = −5.645, df = 145, ***p* < 0.001**	45	8	1.01 (0.97), [−0.30 to 2.48]	*t* = −1.026, df = 86, *p* = 0.308
	Endline	72	0	0.36 (0.92), [−0.30 to 2.48]		43	4	0.80 (0.98), [−0.30 to 2.48]	
**Tank**	Baseline	11	12	1.00 (0.80), [−0.30 to 2.04]	*t* = −1.120, df = 24, *p* = 0.274	10	50	1.52 (0.86), [0.00 to 2.48]	*t* = −1.381, df = 18, *p* = 0.184
	Endline	15	4	0.63 (0.87), [−0.30 to 2.08]		10	9	0.98 (0.89), [−0.30 to 2.48]	
**Tap**	Baseline	14	11	1.14 (0.79), [−0.30 to 2.18]	*t* = −4.086, df = 26, ***p* < 0.000**	9	38	1.54 (1.01), [0.00 to 2.48]	*t* = −2.040, df = 16, *p* = 0.058
	Endline	14	1	0.13 (0.49), [−0.30 to 0.85]		9	3	0.65 (0.82), [−0.30 to 2.48]	

## Supplementary Materials

The following are available online at http://www.mdpi.com/1660-4601/15/8/1616/s1. Section S1: The Regular Monitoring Strategy; Section S2: Water Scheme Upgrades; Section S3: Membrane Filtration Protocol; Section S4: Construction of Field Incubators; Section S5: Water Quality Tests Validity Measurements; Section S6: Detailed Household Survey Results; Section S7: Comparison of Individual Sampling Points; Section S8: Details of Chlorinated Schemes; Section S9: Comparison between Use and Non-Use of Ceramic Candle Filters; Section S10: Detailed Microbial Results; and Section S11: Temporal View of Baseline, Regular Monitoring, and Endline Microbial Data. Scheme-level water quality data are also provided as a supplementary file.

## Abbreviations

CFU	colony forming units
HWTS	household water treatment and safe storage
IWRM	integrated water resources management
MDGs	Millennium Development Goals
NGO	non-governmental organization
NPR	Nepalese Rupee
3R	recharge, retention, and reuse
SDGs	Sustainable Development Goals
Eawag	Swiss Federal Institute of Aquatic Science and Technology
WASH	water, sanitation and hygiene
WSP	water safety plan
WHO	World Health Organization
USD	United States Dollar

## Appendix A

**Table A1:** Sanitary inspection forms for the household water storage containers, piped water taps, reservoir tanks, and sources. Each “yes” answer is scored as 1, and each “no” answer is scored as 0. The risk score equation is given, with the maximum risk score possible being 10.

	Question	Score (yes = 1, no = 0)
	**HOUSEHOLD STORED WATER CONTAINER**	
1	Are the drinking water storage containers used only for storing drinking and cooking water?	
2	Are the drinking water storage containers kept above ground level?	
3	Are the drinking water storage containers’ lids or covers present and in place?	
4	Are the drinking water storage containers sanitary and free from cracks?	
5	Is the area around the drinking water storage containers sanitary?	
6	Are animals prevented from accessing the area around the drinking water storage containers?	
7	Are the taps or utensils used to draw water from the drinking water storage containers sanitary?	
8	Is the water treated in any way before drinking?	
9	Has the water supply been continuous over the past 10 days?	
10	Is the water obtained from only one source?	
	**RISK SCORE = (10 – total # yes answers) =**	
	**PIPED WATER TAP**	
1	Does the tap stand leak?	
2	Is any part of the tap stand cracked or broken?	
3	Is there standing water around the tap stand?	
4	Are there any visible pipe leaks between the tank and the tap stand?	
5	Is the area uphill from the tap stand visibly eroded? (roughly 30m)	
6	Are pipes visibly exposed nearby the tap stand? (roughly 10m)	
7	Is excreta or garbage found within 10 m of the tap stand?	
8	Are there any animals within 10 m of the tap stand?	
9	Is there a sewer or latrine within 10 m of the tap stand?	
10	Has there been discontinuity within the past 10 days at the sample site?	
	**RISK SCORE = (total # yes answers) =**	
	**RESERVOIR TANK**	
1	Are there any visible pipe leaks between the source and the tank?	
2	Is there standing water around the tank?	
3	Is the area uphill from the tank visibly eroded? (roughly 30m)	
4	Are pipes visibly exposed close to the tank? (roughly 10m)	
5	Are excreta, garbage, or animals found within 10 m of the tank?	
6	Is there a sewer or latrine within 10m of the tank?	
7	Has there been discontinuity within last 10 days at the sample site?	
8	Are there signs of leaks around the tank?	
9	Is the tank cracked or damaged?	
10	Are the air vents or inspection covers unsanitary, damaged, or open?	
	**RISK SCORE = (total # yes answers) =**	
	**SOURCE**	
1	Is the water protected from surface contamination (masonry, concrete wall, or spring box)?	
2	Is the structure protecting the source in good condition?	
3	Is there a locked sanitary inspection cover?	
4	Is there a sanitary air vent in the structure?	
5	Is there a sanitary overflow pipe in the structure?	
6	Is there a functional surface water diversion ditch above the source?	
7	Is the source free from contaminating silt or animal excreta?	
8	Is the area around the source properly fenced?	
9	Are animals prevented from entering within 10 m of the source?	
10	Is the area within 10 m of the source free from the presence of latrines?	
	**RISK SCORE = (total # yes answers) =**	

**Table A2:** Baseline and endline water quality at the intervention and control schemes for the households, reservoir tanks, and taps: percentages at guidelines, low risk, and high risk.

Description	Baseline						Endline					
	Households		Tanks		Taps		Households		Tanks		Taps	
	I (*n* = 75)	C (*n* = 45)	I (*n* = 11)	C (*n* = 10)	I (*n* = 14)	C (*n* = 9)	I (*n* = 72)	C (*n* = 43)	I (*n* = 15)	C (*n* = 10)	I (*n* = 14)	C (*n* = 9)
Median [CFU/100 mL]	24	8	12	49.5	10.5	38	0	4	4	8.5	0.5	3
% of samples at the WHO guidelines [0 CFU/100 mL]	17.3	20.0	18.2	0	7.2	0	52.8	23.3	26.7	10.0	50.0	11.1
% of samples at low risk [1–10 CFU/100 mL]	25.3	31.1	27.3	30.0	42.8	33.3	22.2	32.6	40.0	40.0	50.0	66.7
% of samples at higher risk [11-TNTC CFU/100 mL]	57.3	48.9	54.5	70.0	50.0	66.7	25.1	44.2	33.3	50.0	0	22.2

I = Intervention, C = Control.
