# Supplementary material for: Assessing the Impact of a Risk-Based Intervention on Piped Water Quality in Rural Communities: The Case of Mid-Western Nepal
Source: Int J Environ Res Public Health. 2018 Jul 31;15(8):1616. doi: 10.3390/ijerph15081616 (PMC6121632; doi:10.3390/ijerph15081616)
Supplement: Supplementary file 1 [file ijerph-15-01616-s001.pdf]

Assessing the Impact of a Risk-Based Intervention on Piped Water Quality in Rural Communities: The Case of Mid-Western Nepal

Dorian Tosi Robinson, Ariane Schertenleib, Bal Mukunda Kunwar, Rubika Shrestha, Madan Bhatta and Sara J. Marks

Supplementary Materials

Section S1. The Regular Monitoring Strategy

Regular monitoring was performed at each intervention scheme at one source, one reservoir tank, one tap, and one household. All the points were water-connected: the household used water from the corresponding tap, which was connected to the tank and the source that were monitored. The responsibility to select monitoring locations each month was left to the local communities with the help of the local NGO staffs. Each month, the source, tank, tap, and households were rotated. The selection of the households had the condition that the visit should not be planned, and the household not be aware in advance of the upcoming visit. The sources and the tanks were monitored more often than the taps and the households because of their lower number.

Section S2. Water Scheme Upgrades

To improve the safety of the water schemes the following infrastructure upgrades took place: intake improvement with sand and gravel filters, runoff water diversion at the intake, protection work against hazards (landslides, gully, open grazing), minor repairs of the water pipes and structures, and 3R (Recharge, Retention, Reuse) interventions. 3R interventions were expected to show long-term outcomes and were thus only partly operative at the time of writing this paper. For example, plantation work was ongoing at the study endline, and native plants still needed to establish themselves in order to deliver the expected benefits to the local micro-catchment. Additionally, to treat the water and prevent contamination during distribution and handling, two schemes agreed to adopt system-level chlorination practices. Technicians were trained to add a bleach powder solution to the reservoir tank prior to distribution.

Section S3. Membrane Filtration Protocol

To determine the microbial water contamination, the membrane filtration method with the DelAgua kit (DelAgua Group, Lower Fyfield, UK) was used. The protocol has been adapted to the field conditions as follows: (1) The filtration funnel and cup are first dried with clean tissue of all wet residues from the previous processing. (2) It is then disinfected by burning a few drops of methanol inside the metal cup and covering it when almost burnt with the funnel in a loose position to disinfect all surfaces. (3) The system is then air tight and left in this position for 15 minutes to ensure disinfection by the formaldehyde produced through the incomplete combustion of methanol. (4) Hands are at this point disinfected with an ethanol-based disinfectant. (5) A sterile 0.45 µm gridded membrane filter (Merck Millipore, Darmstadt, Germany) is then used for the filtration. It is handled with tweezers that were previously flamed with a lighter and carefully placed on the funnel with the grid facing upwards. (6) All precautions for the sterile technique are taken to ensure that sterilized surfaces are not contaminated. The table surface is disinfected between samples, and the manipulations are done without laying items on the working space unless they are sterilized again. (7) The water sample is poured in the filter funnel up to the 100 mL line. (8) The hand pump is used to create a vacuum and filter the sample. (9) The hands are again sterilized, and a compact dry plate with chromogenic nutrient medium for the detection of coliforms and *E. coli* (Nissui Pharmaceuticals, Tokyo, Japan) is opened and wet with a few drops of sterile water from a baby bottle. This sterile water was previously prepared by boiling a baby bottle in water for more than 5 min and using the

boiling water to fill it. The sterile water is then left to cool to room temperature before use. (10) The membrane filter is then transferred to the compact dry plate with the grid facing upwards using tweezers that were flamed again before use. (11) The compact dry plate is then incubated face down at 35 ± 2 °C for 24 hours in the field incubator.

Section S4. Construction of Field Incubators

A low-cost solar-powered field incubator was developed at Eawag for use in remote field conditions. The main components of the incubator are as follows: an isolation box made of polystyrene walls, an on/off temperature regulator TSM 125 (H-Tronic GmbH, Hirschau, Germany) connected to a temperature probe, a small fan, and heating strips. The incubator maintained a consistent temperature of 35 ± 2 °C throughout the study.

A solar photovoltaic array, a charge controller box and a battery were used to power the incubator. The materials for this setup are readily available in Nepal, as they are commonly used to power house lighting and small appliances in rural areas.

Section S5. Water Quality Tests Validity Measurements

Duplicates: Table S1 summarizes the analysis of duplicates. Overall, most of the duplicates were close to the sample concentration. The mean difference based on the *E. coli* concentration is of 13 CFU/100 mL at the baseline and endline data collection. During regular monitoring, a difference of 5.2 CFU/100 mL was observed. At the baseline and the endline, the maximum difference in *E. coli* between the sample and its duplicate was 293 CFU/100 mL. Similarly, for total coliforms, only two differences were higher than 100 CFU/100 mL (300 and 143 CFU/100 mL, respectively). For regular monitoring and total coliforms, three differences were above 100 CFU/100 mL. The result for *E. coli* was better with a maximum of 28 CFU/100 mL difference between two duplicates.

Table S1. Duplicates statistics: differences between the samples and their duplicates. Units: [CFU/100 mL].

Statistic	Baseline and endline		Regular monitoring	
	E. coli (*n* = 50)	Total coliforms (*n* = 48)	*E. coli* (*n* = 21)	Total coliforms (*n* = 21)
Mean absolute delta	13	16.2	5.2	25.5
CI (95%)	6.1	6.8	1.5	9.1
SD	43.4	48.4	7	41.9
Median (25%; 75%)	2 (0; 6.75)	0 (0; 8.75)	4 (0; 9)	4 (0; 36)
Min delta	0	0	0	0
Max delta	293	300	28	127

Negative controls: Table S2 shows the results of all the negative controls that were carried. Only one negative control tested positive for coliform contamination (2 CFU/100 mL). As all other negative controls were free from contamination, it can be concluded that the quality of the laboratory work in the field conditions was not compromised and that the results are reliable.

Table S2. Results for the negative controls for baseline, regular monitoring and endline.

Sample phase	Negative control free from contamination [%]	Negative control contaminated [%]
Baseline (*n* = 7)	100	0
Regular monitoring (*n* = 23)	95.5	4.5
Endline (*n* = 28)	100	0

Processing time: During the baseline data collection, a subset of samples was processed in more than the two h, as recommended in the protocol. From a total of 120 samples collected from households, 15 were processed in more than 2 h, and 32 were processed in more than 3 h. The maximum time between sampling and processing was 7 h. To verify whether this extended hold time influenced the

results, a Chi square analysis was run (Table S3). There was no statistically significant relationship between the microbial concentration and hold time, indicating that holding times of greater than 3 h did not meaningfully influence results.

Table S3. Chi-Square test of independence for processing time.

Time between sampling and processing	*E. coli* 0 CFU/100 mL vs *E. coli* >0 CFU/100 mL	*E. coli* 0-10 CFU/100 mL vs *E. coli* >10 CFU/100 mL
Exceeded 2 hours	X^2 (1) = 0.846, p = 0.358	X^2 (1) = 0.266, p = 0.0.606
Exceeded 3 hours	X^2 (1) = 0.545, p = 0.46	X^2 (1) = 0.0.003, p = 0.956

Section S6. Detailed Survey Results

Table S4. Household characteristics. All values in percentage [%] if not stated otherwise.

Characteristic	Option	Baseline (*n* = 120)	Endline (*n* = 115)
Respondent	Male head	42.5	27.5
	Female head	37.5	43.3
	Other male	3.3	6.7
	Other female	16.7	18.3
Age of interviewee (years)	-	18–78 (M = 41, SD = 14)	18–82 (M = 40, SD = 15)
Household population (number)	-	2–16 (M = 6.7, SD = 2.6)	1–15 (M = 6.5, SD = 2.3)
Children age 6–18 per household (number)	-	M = 2.1, SD = 1.4	M = 2, SD = 1.3
Children age <5 per household (number)	-	M = 1, SD = 1	M = 0.8, SD = 0.9
Highest level of education of interviewee	Illiterate	36	21
	Literate	26.5	38
	Primary	9	18
	Secondary	16	14
	University/college	11.5	9
	Post-graduate	1	0
Occupations of the household*	Farming/agriculture	99	99
	Job/service	50	20
	Business	26	16
	Abroad/foreign employment	0	1
	Daily labor in their own community	11	6
	Seasonal employment in India	46	50
	Seasonal employment in Nepal	4	1
	Other	3	0
Involvement of any member of the household to a leadership position	No	97	88
	Chair person	0	3
	Mayor	1	0
	Deputy mayor	0	0
	Member of local body	0	0
	Other	1	0
Ethnicity of the household	Dalit	20	20
	Janjati	0	0
	Other	80	80
Main concern of the household	Health and healthcare services	5	12
	Sanitation	1	6
	Water supply services	39	13
	Transportation and roads	4	11
	Agriculture	5	20

	Electricity	20	24
	Jobs	23	3
	Education	3	8
	Security and crime	0	1
	Other	0	0
	Do not know	0	0
Household monthly expenses (NPR)	-	2000–42,000(M = 10,370, SD = 7920)	1550–50,000(M = 10,610, SD = 7800)

* Multiple answers possible.

Table S5. Household observations. All values in percentage [%].

Survey Question	Options	Intervention		Control	
		Baseline (*n* = 75)	Endline (*n* = 72)	Baseline (*n* = 45)	Endline (*n* = 43)
What are the walls made of?	Stone/Brick/Cement/concrete blocks	26.7	1.4	15.6	0
	Wood/mud	73.3	95.8	84.4	97.7
	Metal	0	2.8	0	2.3
	Sticks	0	0	0	0
	Straw	0	0	0	0
	Other	0	0	0	0
What is the roof made of?	Metal/sheet metal	58.7	75	35.6	44.2
	Thatch/branches	28	13.9	48.9	14
	Cement/concrete	0	0	0	0
	Wood	0	0	0	0
	Tiles	6.7	6.9	2.2	4.7
	Mud	2.7	1.4	13.3	14
	Plastic	0	0	0	0
	Other	4	2.8	0	23.3
What is the floor made of?	Cement/concrete	5.3	1.4	2.2	2.3
	Tile	0	0	0	0
	Wood	4	2.8	0	0
	Mud/sand/dirt	86.7	95.8	93.3	97.7
	Other	4	0	4.4	0
What type of toilet is present?	Flush toilet in home	1.3	0	2.2	2.3
	Improved latrine private	92	97.2	88.9	93
	Improved latrine shared	0	1.4	2.2	4.7
	Improved latrine public	0	0	2.2	0
	Unimproved latrine private	6.7	0	4.4	0
	Unimproved latrine shared	0	0	0	0
	Unimproved latrine public	0	1.4	0	0
	No facility	0	0	0	0
	Other	0	0	0	0
Does the household have electricity connection?	Yes, working	86.7	94.4	88.9	90.7
	Yes, not working	4	2.8	0	2.3
	No	9.3	2.8	11.1	7

Table S6. Household handwashing practices. All values in percentage [%].

Survey Question	Options	Intervention		Control	
		Baseline (*n* = 75)	Endline (*n* = 72)	Baseline (*n* = 45)	Endline (*n* = 43)
When did the interviewee wash his hands	After going to toilet	99	99	91	100
	Before eating	93	99	98	95
	Before cooking	68	82	67	63

the previous day? *	After cleaning baby's bottom	45	31	62	21
	After field work	60	92	73	86
	No critical times mentioned	20	0	29	12
Frequency of soap use during the previous day handwashing events?	Always	43	62.5	80	60.5
	Sometimes	56	37.5	20	39.5
	Never	1	0	0	0
Type of handwashing facility?	Handwashing station with faucet	65.5	83.5	84.5	81.5
	Bowls/jugs	20	8.5	4.5	11.5
	Tap	8	3	4.5	4.5
	Other	0	3	4.5	2.5
	No facility	6.5	3	2	0

* Multiple answers possible.

Table S7. Drinking water quality perception. All values in percentage [%].

Subject	Options	Intervention		Control	
		Baseline (*n* = 75)	Endline (*n* = 72)	Baseline (*n* = 45)	Endline (*n* = 43)
Water taste perception *	Good	98.5	93.0	98.0	83.5
	Salty	1.5	0	0	2.5
	Chlorine	0	5.5[1]	0	2.5
	Rusty/metallic	0	0	0	2.5
	Soil/dirt	0	0	0	0
	Varies from rainy to dry months	2.5	0	4.5	2.5
	Other	0	0	0	0
	Do not know	0	1.5	0	9.5
Water smell perception *	No smell	100.0	93.1	100.0	88.4
	Smells bad/funny	0	5.6 [1]	0	7.0
	Do not know	0	1.5	0	4.7
Water color perception *	Good/clear	92.0	93.0	95.5	79.0
	Cloudy	1.5	5.5	0	4.5
	Brown	0	0	0	2.5
	Varies from rainy to dry months	8.0	1.5	20.0	0
	Other	1.5	0	0	0
	Do not know	2.5	0	0	14.0
Safety perception	Generally safe	89.5	98.5	84.5	79
	Somewhat unsafe	6.5	1.5	11.0	14.0
	Very unsafe	0	0	0	2.5
	Varies from rainy to dry months	2.5	0	4.5	0
	Do not know	1.5	0	0	4.5
Do you treat your water in any way?	Yes	69.5	100.0	86.5	90.5
	No	30.5	0	13.5	9.5
If you treat the water, how? *	Chlorine	0	12.5	0	2.5
	Screening/filtering with a cloth	0	0	2.5	2.5
	Ceramic candle filter	88.5	97.0	97.5	95.0
	Silver coated filter	0	1.5	0	2.5
	Coagulation/settlement	0	0	0	0
	UV/SODIS	0	0	0	0
	Boiling	17.0	7.0	7.5	15.5
	Other	0	0	0	0
	Do not know	0	0	0	0
How often do you treat it?	Always	77.5	91.5	89.5	84.5
	Almost always	11.5	8.5	0	13.0
	Sometimes	4.0	0	2.5	2.5
	Rarely	7.5	0	5.0	0
	Do not know	0	0	2.5	0

* Multiple answers possible; [1] Considering only chlorinated schemes, chlorine taste is reported by 15% of the 29 households concerned. Smell perception as bad/funny is 14% for chlorinated schemes.

Table S8. Water supply characteristics. All values in percentage [%].

Survey Question	Options	Intervention		Control	
		Baseline (*n* = 75)	Endline (*n* = 72)	Baseline (*n* = 45)	Endline (*n* = 43)
What are the water sources used by the household? *	Private tap	17.5	18	2	0
	Public tap	85.5	82	98	97.5
	Rainwater harvesting	0	1.5	0	0
	Protected source	30.5	37.5	9	14
	Open source	8	1.5	2	4.5

What is the main water source?	Private tap	17.3	18	2	0
	Public tap	81.3	80.5	98	100
	Rainwater harvesting	0	0	0	0
	Protected source	1.3	1.5	0	0
	Open source	0	0	0	0
Is the drinking water source functional?	Functioning well	85.5	86	80	88.5
	Functioning not well	14.5	14	20	11.5
	Not functioning	0	0	0	0
Are you confident about repairs done within a week?	Very confident	80	82	71	90.5
	Somewhat confident	14.5	14	24.5	9.5
	Not confident at all	5.5	1.5	4.5	0
	Do not know/no answer	0	0	0	0
Can you get help by the VMW?	Yes	86.5	95.8	100	100
	Maybe	13.5	2.8	0	0
	No	0	0	0	0
	Do not know/no answer	0	1.4	0	0
Do you know where to find spare parts for repairs?	Yes	49.3	63.9	44.4	39.5
	Maybe	6.7	5.6	4.4	4.7
	No	37.3	25	44.4	48.8
	Do not know/no answer	6.7	5.6	6.7	7
In the past 6 months, was the water not available for more than a week?	Yes	10.7	9.7	8.9	0
	No	89.3	87.5	88.9	100
	Don't know/no answer	0	2.8	2.2	0
How is the reparation paid for?	Water tariff	89.3	84.7	91.1	100
	Users contribute money as	0	12.5	8.9	0
	needed	0	0	0	0
	Users contribute in kind	1.3	1.4	0	0
	regularly	0	0	0	0
	In kind as needed	8	0	0	0
	Someone else pays	2.7	1.4	0	0
	No one pays				
	Don't know/no answer				
Do you think your water source will be functional in a year?	Yes	85.3	87.5	88.9	100
	No	8	0	6.7	0
	Don't know/no answer	6.7	12.5	4.4	0

* Multiple answers possible.

Table S9. Water supply characteristics. All values in percentage [%] unless stated otherwise.

**Survey Question**	**Option**	**Baseline (n = 120)**	**Endline (n = 115)**
Involvement of any member of the household to the water project? *	No	68	66
	Member of water users and sanitation	0	23
	committee	8	3
	Member of WSP team	3	2
	Village maintenance worker	2	3
	Female community health volunteer	1	10
	Women tap stand care taker	2	4
	Other		
How often does the water and sanitation users' committee meet together?	Monthly	76	76
	Bi-monthly	2	1
	Once every 3 months	3	2
	Once every 6 months	0	0
	Once per year	0	0
	As needed	7	18
	Never	3	0

	Do not know	9	3
How often does the water and sanitation users' committee meet with the community?	Monthly [1]	53	75
	Bi-monthly	5	1
	Once every 3 months	3	2
	Once every 6 months	1	0
	Once per year	8	2
	As needed	17	17
	Never	2	1
	Do not know	11	3

* Multiple answers possible; [1] During baseline, 60% of intervention and 40% control schemes respondents indicated monthly WUSC meetings with the community. At endline, the percentage increased with 79% and 67%, respectively.

Table S10. Additional survey questions asked within intervention schemes. All values in percentages [%] if not otherwise stated.

Survey Question	Options	Endline
Have you heard about the WSP in your community? (*n* = 72)	Yes	87.5
	No	12.5
Did you participate to the WSP preparation? (*n* = 63)	Yes	54
	No	46
What was your task? (*n* = 34)	Member of the WSP task force	23.5
	Voluntary involved	76.5
	Other	0
What activities were done after WSP preparation? (*n* = 63)	Chlorination	44.4
	Installation of intake filter	90.5
	Regular WUSC meetings	49.2
	Regular sanitary inspection	61.9
	Cleaning of structures	52.4
	Fixing broken parts	42.9
	Other	0
Do you know about the microbial water quality testing laboratory? (*n* = 72)	Yes	93.1
	No	6.9
Did you notice water quality testing at your household, tap, or tank? (*n* = 72)	Yes, at the tap	75
	Yes at the tank	51.4
	Yes at the household	88.9
	No	0
Did you receive the results from the tests? (*n* = 72)	Yes	70.8
	No	29.2
From whom? (*n* = 51)	Lab staff	35.3
	Sampler	21.6
	WUSC member	62.7
	Visited the lab on my own	7.8
	NGO staffs	72.5
	Other	0
How was the result? (*n* = 51)	Good	62.7
	Bad	37.3
	Don't know	0
What activity did you start after learning about the bad quality? (*n* = 19)	Use other tap	0
	Treatment of water	100
	Nothing	0
	Other	0
What treatment did you start? (*n* = 19)	Boiling	15.8
	Ceramic candle filter	100
	Chlorination	0
	Silver coated filter	0
	Screening/filtering with a cloth	0
	UV/Sodis	0
	Do not know	0
	Other	0
Would you ask for water quality tests on your own? (*n* = 72)	Yes	95.8
	No	4.2
How much would you pay? (*n* = 69)		0–500 NPR (M = 74,SD = 87)
Are you a member of the WUSC? (*n* = 72)	Yes	12.5
	No	87.5
Do you know about the results of monthly monitoring of WQ? (*n* = 9)	Yes	100
	No	0
Who have informed you about these results? * (*n* = 9)	Lab staff	44.4
	Water sampler	44.4
	NGO staff	88.9

	Visited the lab on my own	11.1
	Other	0
What do you do with the results? (n = 9)	Discuss among the WUSC	88.9
	Inform the community	33.3
	Take action for improvement	44.4
	Nothing	0
	Other	

* Multiple answers possible.

Table S11. Household samples characteristics. All values in percentage [%].

Survey Question	Options	Intervention		Control	
		Baseline (n = 75)	Endline (n = 72)	Baseline (n = 45)	Endline (n = 43)
How is the visual aspect of the sample you took?	Clear	100	97.2	95.6	81.4
	Debris present	0	2.8	2.2	9.3
	Somewhat turbid	0	0	2.2	9.3
	Very turbid	0	0	0	0
Did the sampled water receive a treatment?	Yes, at system level	0	0	4.4	0
	Yes, at household level	58.7	25	75.6	86
	Yes, at system and household	2.7	75	2.2	0
	level	37.3	0	17.8	14
	No	1.3	0	0	0
	Don't know				
Where is the water sampled from? [2]	Ceramic candle filter	57.3	98.6	77.8	81.4
	Gagri	22.7	1.4	17.8	7
	Jerrycan	4	0	0	0
	Gallon	14.7	0	4.4	11.6
	Bucket	1.3	0	0	0
Is the water chlorinated? [1]	Yes	0	41.1	0	0
	No	100	58.9	100	100

[1] The two chlorinated scheme account for all the samples reported as chlorinated here. All of the samples of these two schemes are reported as chlorinated. No test was performed to measure chlorine levels; only the chlorination practice at the scheme level was reported. [2] This result is extracted from the field inspection forms and not from the household survey answers.

Section S7. Comparison of Individual Sampling Points

Table S12 shows the percentage of samples at better or identical water quality at the endline as compared with the baseline. Each sampling point was compared before and after the intervention to compute this result.

Table S12. Percentage of samples with better or identical water quality at the endline as compared with the baseline.

Location	Intervention	Control
Household (n = 72; 43)	83.3	53.5
Tank (n = 11; 9)	45.5	55.6
Tap (n = 14; 9)	92.9	66.7

There is a significant association at the household level between better or identical water quality at the endline compared with the baseline and whether or not the scheme received intervention (χ^2 (1) = 11.94, p = 0.001). Based on the odds ratio, the odds of better or identical water quality at the household in an intervention scheme are 4.35 times higher than in a control scheme.

At the tanks and the taps, the Chi square test assumptions are violated. The likelihood ratio can be used in this case, but no significant associations are observed at any point (Tank: LR = 0.202, p = 0.653; Tap: LR = 2.592, p = 0.107).

Section S8. Details of Chlorinated Schemes

Two drinking water schemes in the intervention area adopted chlorination activities at the scheme level as part of the upgrades of the system. Chlorination was done by pouring a bleach solution into the reservoir tanks of schemes 1 and 5 and waiting until the tanks filled and mixed thoroughly before distribution to taps. At scheme 1, the water quality was very good at the household and tap level (−0.3 Log10 *E. coli* CFU/100 mL for both), which were water samples that likely had residual chlorinate. In this scheme, the water quality at the reservoir tank was worse (1.71 Log_{10} CFU *E. coli*/100 mL), because samples were drawn at the tank inlet (above the chlorinated supply). Additionally, household samples from scheme 1 had the lowest total coliform concentration with 78.6% of them being free from any coliform. Similarly, no coliforms were counted in the tap samples, while the tank samples had 300 total coliform CFU/100 mL.

During the endline, it rained during the day and night before the samples were collected in scheme 5, and chlorine was likely washed out due to the overflow of the tank. The water quality at the household level for this scheme was not as good (1.07 Log_{10} CFU *E. coli*/100 mL). At the tank and the tap, the mean contamination concentrations were 1.73 and 0.06 Log10 CFU *E. coli*/100 mL, respectively. Again, chlorination only affects the quality at the taps or the households, as the water at the tank inlet is not yet chlorinated. The total coliform concentration at scheme 5 follows a similar trend as *E. coli* measurements. The tank samples had 300 CFU/100 mL total coliforms. The taps only had a few (0, 1, and 17 CFU/100 mL). Thus, the multibarrier approach with source protection, chlorination, and household treatment was not sufficient to provide safe drinking water in the case of scheme 5.

Section S9. Comparison between Use and Non-Use of Ceramic Candle Filters

The use of filters increased throughout the study, and better water quality at the household level was observed from the baseline to endline period. Based on all the household stored water samples collected, simple linear regression indicated a mean difference in *E. coli* contamination of 0.837 Log_{10} CFU/100 mL between samples taken from a filter and from another type of container (SE = 0.147, t = -5.689, p <0.001, n = 258). There was a significant correlation between better water quality and the use of household ceramic filters at the baseline (p = 0.014), regular monitoring (p = 0.013), and the endline (p = 0.001).

Additionally, a Mann–Whitney U test indicated that the median *E. coli* contamination measured during the endline at the household level was significantly lower for samples from filters (Mdn = 1 CFU/100 mL) than from other types of containers (Mdn = 100 CFU/100 mL), U = 180.5, p = 0.001, r = -0.3. A similar result was observed at the baseline. The Supplementary Materials Tables S15 and S16 report the detailed water quality results at the baseline and endline for filter and non-filter samples.

Section S10. Detailed Microbial Results

Table S13. Intervention schemes median *E. coli* contamination at the baseline and endline.

**Location**	**Baseline median [CFU/100 mL]**	**Endline median [CFU/100 mL]**	**Mann–Whitney U test (asymp. sig. (2-tailed))**
Household (n = 75; 72)	24	0	U = 1360, p = 0.000, z = −5.297, r = −0.44, medium-size effect
Tank (n = 11; 15)	12	4	U = 64.5, p = 0.347
Tap (n = 14; 14)	10.5	0.5	U = 28.5, p = 0.001, z = −3.234, r = 0.61, large-size effect

Table S14. Control schemes median *E. coli* contamination at baseline and endline.

**Location**	**Baseline median [CFU/100 mL]**	**Endline median [CFU/100 mL]**	**Mann–Whitney U test (asymp. sig. (2-tailed))**
Household (n = 45; 43)	8	4	U = 850.5, p = 0.325
Tank (n = 10; 10)	49.5	8.5	U = 32.5, p = 0.185

Tap (n = 9; 9)	38	3	U = 21.5, p = 0.091

Table S15. Baseline ceramic filters water quality (*E. coli* contamination).

	With Filter (n = 78)	Without Filter (n = 42)
Mean *E. coli* concentration [Log_{10} CFU/100 mL] (SD), [Range]	1 (1), [−0.3 to 2.48]	1.46 (0.9), [−0.3 to 2.48]
Median [1] *E. coli* concentration [CFU/100 mL]	12.5	31.5
% at the WHO guidelines (0 CFU/100 mL)	23	5
% at low risk (1–10 CFU/100 mL)	25.5	28.5
% at higher risk (11-TNTC CFU/100 mL)	51.5	66.5

[1] Mann–Whitney U (asymp. Sig. (2-tailed)): U = 1195, p = 0.014, z = −2.448, r = −0.22, small effect size

Table S16. Endline ceramic filters water quality (*E. coli* contamination).

Statistic	With Filter (n = 106)	Without Filter (n = 9)
Mean *E. coli* concentration [Log_{10} CFU/100 mL] (SD), [Range]	0.44 (0.93), [−0.3 to 2.48]	1.5 (0.93), [0–2.48]
Median [1] *E. coli* concentration [CFU/100 mL]	1	101
% at the WHO guidelines (0 CFU/100 mL)	45.3	0
% at low risk (1–10 CFU/100 mL)	25.5	33.3
% at higher risk (11-TNTC CFU/100 mL)	29.2	66.7

[1] Mann–Whitney U (asymp. Sig. (2-tailed)): U = 180.5, p = 0.001, z = −3.212, r = −0.3, medium effect size

Section S11. Temporal Representation of Baseline, Regular Monitoring, and Endline Data

Figures S1–S3 show a temporal representation of the median *E. coli* contamination at the baseline and endline periods, with the regular monitoring points in between for the households, tanks, and taps. A somewhat downward trend in the contamination level between the baseline and endline was observed at the household level. Nevertheless, major spikes were observed for some communities during the regular monitoring period (August–December). Among reservoir tanks, the trend was extremely variable with frequent large spikes, indicating a point of vulnerability in the overall system. Also, the tank water quality did not appear to align with what was observed at the household level, suggesting that household hygiene behavior played the important role in determining the water quality at the point of consumption and therefore overall health risk. At the taps, a general downward trend was observed, but (like tanks) spikes in the contamination were seen in certain months, indicating a system vulnerability and health hazard. Overall, regular monitoring indicated a trend of improved drinking water quality but without the guarantee of safety during all months.

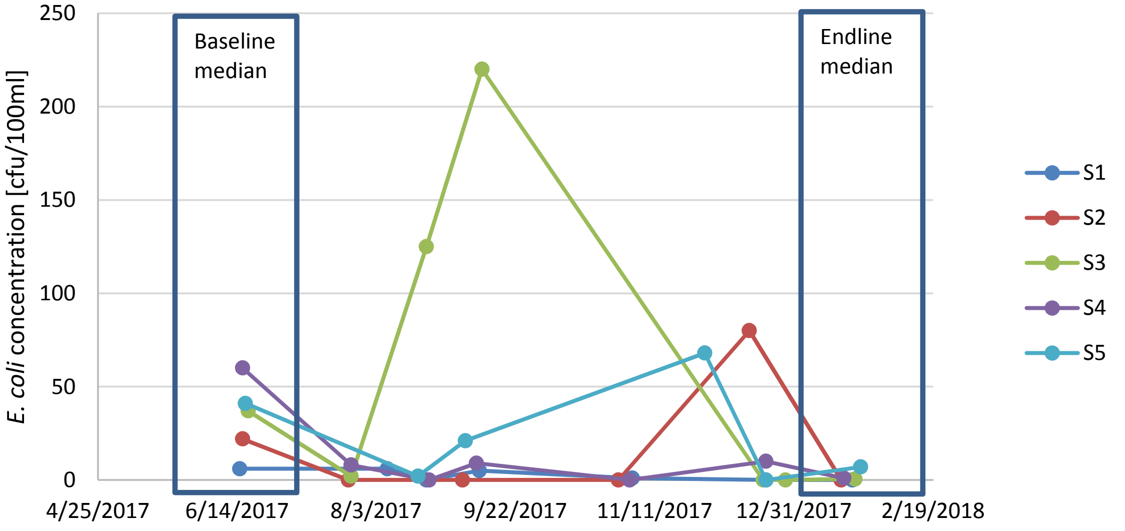

Figure S1. Baseline and endline median *E. coli* contamination and regular monitoring data for the household stored water containers within schemes S1–S5. During the regular monitoring period (August–December), only one household stored water container was sampled per scheme.

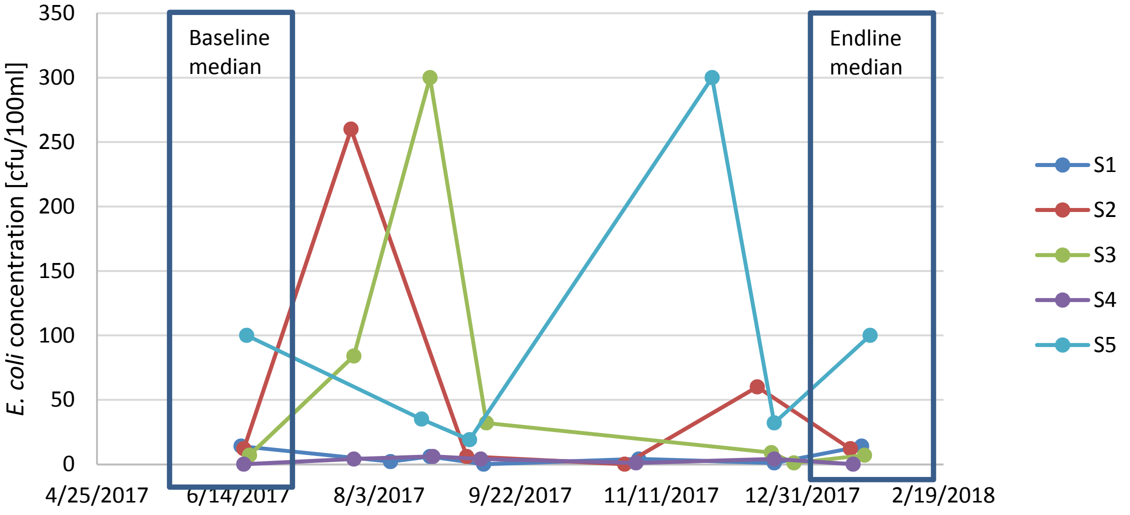

Figure S2. Baseline and endline median *E. coli* contamination and regular monitoring data for reservoir tanks within schemes S1–S5. During the regular monitoring period (August–December), only one tank was sampled per scheme.

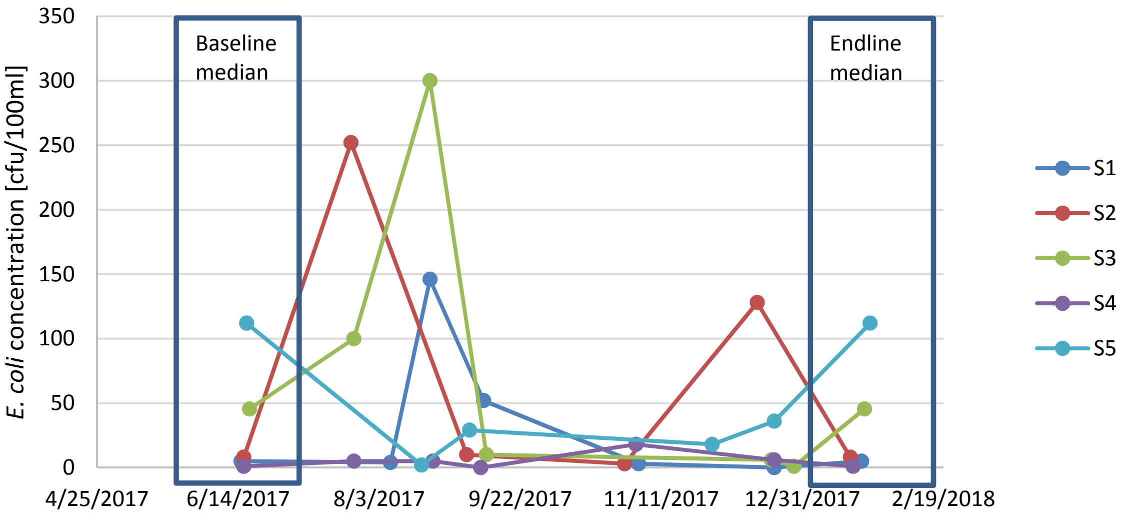

Figure S3. Baseline and endline median *E. coli* contamination and regular monitoring data for taps within schemes S1–S5. During the regular monitoring period (August–December), only one tap was sampled per scheme.

Bivariate Comparisons (Median Approach)—Additional Information

Figure S4 shows the median *E. coli* concentrations at each sampling point for the intervention and control schemes at the baseline and endline periods.

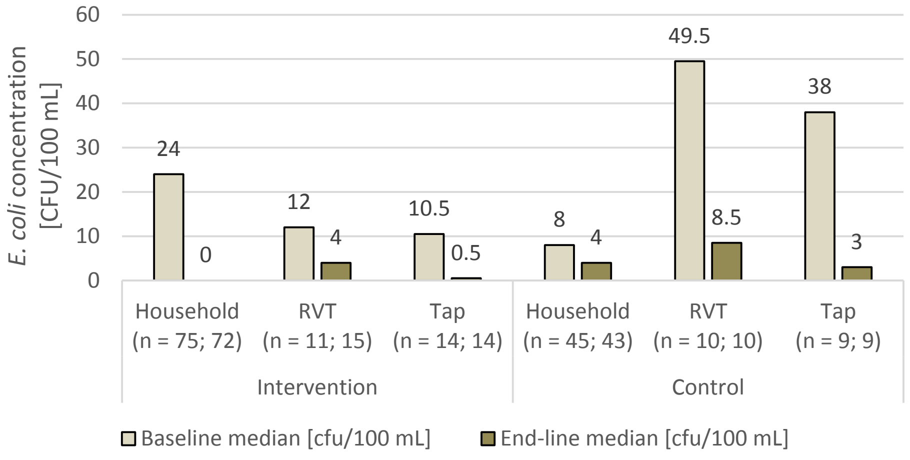

Figure S4. Median *E. coli* contamination at the baseline and endline periods for the intervention and control schemes at each sampling point.
